# Mechanotransduction in intervertebral discs

**DOI:** 10.1111/jcmm.12377

**Published:** 2014-09-30

**Authors:** Tsung-Ting Tsai, Chao-Min Cheng, Chien-Fu Chen, Po-Liang Lai

**Affiliations:** a Department of Orthopaedic Surgery, Spine Section, Chang Gung Memorial Hospital and Chang Gung University College of MedicineTaoyuan, Taiwan; b Institute of Nanoengineering and Microsystems, National Tsing Hua UniversityHsinchu, Taiwan; c Graduate Institute of Biomedical Engineering, National Chung Hsing UniversityTaichung, Taiwan

**Keywords:** mechanotransduction, intervertebral discs, physical forces, gene expression, biochemical activities, protein synthesis

## Abstract

Mechanotransduction plays a critical role in intracellular functioning—it allows cells to translate external physical forces into internal biochemical activities, thereby affecting processes ranging from proliferation and apoptosis to gene expression and protein synthesis in a complex web of interactions and reactions. Accordingly, aberrant mechanotransduction can either lead to, or be a result of, a variety of diseases or degenerative states. In this review, we provide an overview of mechanotransduction in the context of intervertebral discs, with a focus on the latest methods of investigating mechanotransduction and the most recent findings regarding the means and effects of mechanotransduction in healthy and degenerative discs. We also provide some discussion of potential directions for future research and treatments.

- Introduction
- Investigating mechanotransduction
- Mechanotransduction in differing tissue types
- The anatomy of the intervertebral disc
- The effects of mechanical stress on intervertebral discs
- Cyclic tensile stress *versus* compressive stress
- Mechanotransduction in healthy and degenerated discs
- Mechanoreceptors in intervertebral discs
- Gene expression in intervertebral discs
- Conclusion

## Introduction

**Fig. 1:**
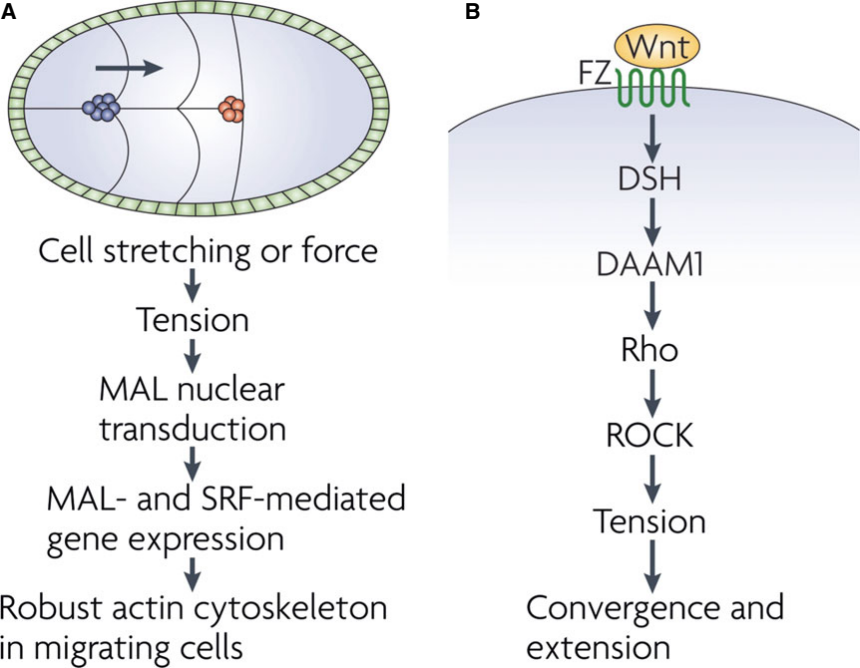
(**A**) During the border cell migration process of *Drosophila melanogaster* oogenesis, follicle cell migration proceeds along the midline of the egg chamber (the cells in blue are the migrating cells, and the cells in red indicate their final positions). The tension created as these cells are stretched or subjected to external force during their migration causes nuclear translocation of MAL, the SRF cofactor. As a result, the nuclear MAL and SRF are able to regulate the expression of various genes, including those needed for cytoskeletal integrity. The illustrated model is suggested to explain how cells form and maintain a strong actin cytoskeleton during their migration. (**B**) The planar cell polarity pathway, which is also called the non-canonical Wnt pathway, regulates various morphogenetic movements that result in spatial cell and tissue rearrangements during extension and convergence. When the Frizzled (Fz) receptor is binded by Wnt, Disheveled (Dsh) is then activated, which in turn activates Daam1, which in turn results in RhoA activation and ROCK-generated cellular tension and contractility. (Figure adapted from Wozniak M, Chen CS. Mechanotransduction in development: a growing role for contractility. *Nat Rev Mol Cell Biol*. 2009; 10: 34–43.)

In cells, mechanotransduction is the means by which physical forces, such as stretching, compression and shear stress, are translated into biochemical impulses and signals [1]. These biochemical changes can include adjustments to intracellular concentrations of enzymes and elements (potassium and calcium, for example), as well as the activation of various signalling pathways, each of which may, in turn, result in changes to both cellular and extracellular structures. Through these various mechanotransduced pathways, the mechanical forces of the three-dimensional environments in which cells exist contribute to the regulation of even the most fundamental of cellular processes, including protein synthesis, adhesion, proliferation and apoptosis, among others (Fig. 1) [2].

Because of the critical role that mechanotransduction plays in such processes, any substantial disruption of normal mechanotransduction can have powerful effects; defective mechanotransduction has been implicated in a variety of diseases [3]. For example, abnormal mechanotransduction in cardiac or skeletal muscle can result in cardiomyopathy or muscular dystrophy [4–6]. A variety of other tissues can incur similarly deleterious effects, including bone [7], lung tissue [8–10] and the tissues of the central nervous system [11,12], to name only a few examples.

In the light of the complex interplay between cellular functions and the extracellular surroundings of cells described above, it is not terribly surprising that pathological consequences can also occur purely as a result of extracellular physical changes. That is, even when mechanotransduction-related processes within cells are functioning normally, disturbances in the physical inputs they receive from their extracellular environs can subsequently cascade to produce pathological results. The loss of bone mass experienced by astronauts living for extended periods in reduced gravity is one interesting example of such a disturbance and its effects [13]. The minimal amount of gravitational force exerted on the astronauts (relative to the gravitational force on the earth's surface), causes disruptions to normal mechanotransductive processes in bone tissues that lead to diminished tissue production and reduced bone mass [13]. A far more common but not unrelated example is the atrophying of muscle tissue that occurs in patients who become bedridden or otherwise immobilized for extended periods of time [14].

## Investigating mechanotransduction

**Fig. 2:**
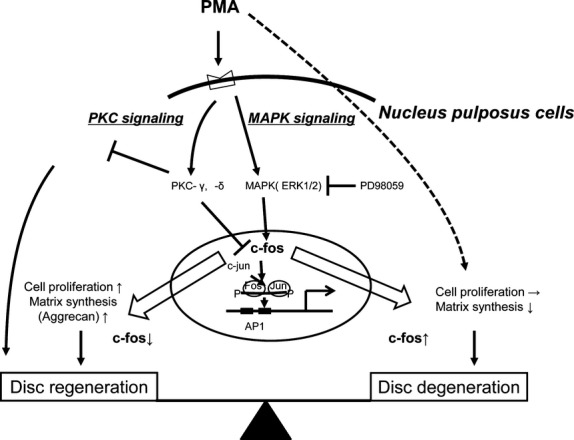
A suggested model for the regulation of c-fos expression via MAPK signaling and PKC signaling in NP cells. (Figure adapted from Yokoyama K, Hiyama A, Arai F, *et al*. C-Fos regulation by the MAPK and PKC pathways in intervertebral disc cells. *PLoS ONE*. 2013; 8: e73210.)

Understanding mechanotransduction can be difficult because of the sheer complexity of the systems involved. To take an example directly relevant to the topic of this review, Figure 2 provides a model of how a mitogen-activated protein kinase (MAPK) mechanotransduction signalling pathway (which also involves phorbol 12-myristate 13-acetate and protein kinase C) may serve to regulate c-fos expression in nucleus pulposus cells in the context of both intervertebral disc (IVD) degeneration and regeneration [15]. For many readers, both the preceding sentence and Figure 2 will likely seem fairly difficult to decipher. However, in the course, of this review, we hope to break the complex process of mechanotransduction down into more simple and understandable components, to make Figure 2 and similar models of the process substantially easier to understand.

As noted above, the term mechanotransduction refers to the process by which cells translate external physical forces into internal biochemical activity. In the context of living tissues, however, that definition can seem overly simplistic insofar as it glosses over a great many details. For one thing, cells do not exist, *in vivo*, in isolation (outside of unicellular organisms, that is), and the mechanotransduced activities in one cell can cause that cell, in turn, to affect others—whether in terms of adhesion, proliferation, apoptosis, *etc*.—in a chain of action and reaction that can often loop back to the first cell in a continuing cycle. Meanwhile, cells also exist in extracellular matrices (ECM), which can themselves be affected by, propagate and effectively originate mechanotransductive activity. In short, the complexities of mechanotransduction make it difficult to isolate and understand the myriad factors involved.

For example, while compression and hypo-osmotic stretching have previously been used to elucidate mechanotransduction in dorsal root ganglion sensory neurons [16,17], and glass pipettes and pressure jets have been used to measure the activation of nerve terminals subjected to pressure [18,19], these non-specific methods of mechanical neural activation failed to fully account for the structural interactions, such as focal adhesions that also occur in the tissues tested [20].

**Fig. 3:**
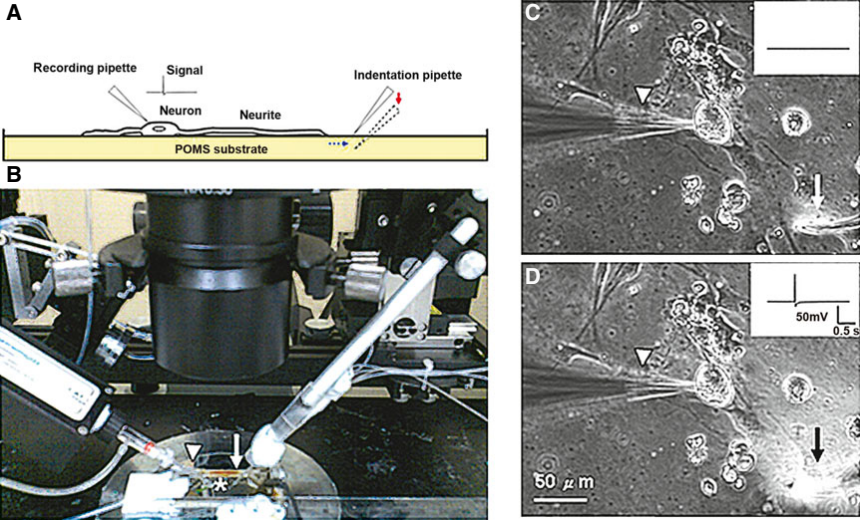
An experimental set-up involving the use of elastomeric substrates to analyse mechanotransduction in neurites. (**A**) Schematic diagram illustrating the mechanical stretching forces applied to neurites in the recording chamber. A recording pipette was utilized to record DRG neurons cultured on a polydimethylsiloxane (PDMS) substrate. A glass pipette was used to indent the PDMS substrate, thereby generating force on the neurites. The substrate transmitted the force applied by the indenting pipette to the cell, and a whole-cell patch clamp set-up was used to record the resulting signal from a given DRG neuron. (**B**) A photograph of the system utilized to record the stretch-evoked AP response from the neurites. The recording itself was conducted in a recording chamber (indicated by the asterisk), which was constantly perfused with ACSF. A pre-amplifier was attached to a recording pipette (white arrowhead) that was connected directly to the neuron. Each neurite examined was stretched by the indentation force from the pipette (white arrow), and the indentation itself was directed using a micromanipulator. (**C**) Placing the indentation pipette (white arrow) on the surface of the PDMS substrate near the neurite was not sufficient to generate any AP (inset). (**D**) As the PDMS became indented by the pipette through vertical displacement (micromanipulator), however, a change in the intensity of the differential interference contrast (DIC) image at the location of the pipette was observed (black arrow). This indentation exerted a force on the attached cell, which in turn evoked an AP (inset). Doi: 10.1371/journal.pone.0004293.g001. (Figure adapted from Lin YW, Cheng CM, LeDuc PR. Understanding sensory nerve mechanotransduction through localized elastomeric matrix control. *PLoS ONE*. 2009; 4: e4293.).

More recently, however, a number of studies have used elastomeric matrices that can effectively be adjusted to simulate the actual physiological conditions of living tissues, providing greater insight into mechanotransduction mechanisms and processes [21]. For example, Lin *et al*. used polydimethylsiloxane (PDMS) coated with ECM as an elastic substrate to determine how mechanical stimulation of the ECM and chemical disruption of cytoskeletal structures affect mechanotransduction in sensory neurons (Fig. 3) [22]. A related study by Cheng *et al*. cultured dorsal root ganglia neurons on PDMS substrates of varying rigidity and found a clear relationship between substrate elasticity and a host of outcome such as neural growth rates, the distribution of proteins in the different substrates and the responses of structural proteins [23]. With a similar focus, a study by Engler *et al*. provided information on how differing degrees of matrix elasticity/rigidity can effectively determine the phenotypes of naïve mesenchymal stem cells—that is whether they ultimately develop into bone, muscle or nervous tissues [24]. Meanwhile, Sniadecki *et al*. developed a system of elastomeric microposts embedded with cobalt nanowires to apply nanoscale traction forces to cells and then measure the resulting cellular responses, while a study by Munevar *et al*. used traction force microscopy and peptide-induced stress to investigate the mechanical interactions of fibroblasts migrating on a substrate, determining that leading edge and trailing edge adhesions exhibit distinct mechanical interactions [25,26]. Finally, Bellin *et al*. used elastomeric substrates to identify syndecan-4 as a non-integrin transmembrane protein that can also initiate mechanotransduction (an important finding insofar as the bulk of previous research has looked at the integrins as the main transmembrane protein means of starting mechanotransduction [27].

In addition to such research using elastomeric substrates, various other techniques have also proven useful for the study of mechanotransduction. Among the more fruitful thus far is magnetic twisting cytometry, a technique which relies on the magnetic signal-induced twisting of membrane-bound magnetic microbeads, the resulting rotations of which are measured using a magnetometer [28] (some of the specific findings derived from this technique are discussed below).

## Mechanotransduction in differing tissue types

Of course, even as such groundbreaking research allows researchers to gain a fuller understanding of how mechanotransduction functions in living tissue, it is important to remember that the tissue types within the bodies of complex organisms can vary substantially, such that mechanotransduction in one type of tissue may be qualitatively different than that in another tissue type.

For example, the unique functional and physical characteristics of IVDs, which link the vertebral bodies to constitute the main joints of the spine, indicate that mechanotransduction plays a particularly prominent role in their normal function and, as the discs age, their dysfunction. A purpose of the discs is to transmit physical forces, namely, physical forces resulting from muscle movement and the weight of the body, to the spinal column, with the discs providing the tension, flexion and bending capacity that give the spine its flexibility [29]. In effect, each disc functions much like a small shock absorber in a car, taking in the physical forces delivered to the spine and protecting the bony vertebrae above and below it by keeping them separate.

## The anatomy of the IVD

Structurally speaking, IVDs consist of two main parts: the annulus fibrosus and the nucleus pulposus. The former, a thick outer ring consisting of several layers of fibrocartilage, surrounds the latter, which forms an inner core and has a softer, more gelatinous texture. A relatively minor but nonetheless distinct third part of IVDs are the cartilage endplates on the top and bottom of each disc that provide an interface between the given disc and the vertebrae above and below it, effectively sandwiching both the inner nucleus pulposus region and outer annular fibrosus ring.

The primary proteoglycan of the disc is aggrecan, which is the main means by which the disc maintains tissue hydration. The nucleus has a much higher concentration of these proteoglycans than the annulus—this means that the nucleus has a much higher degree of hydration, which is itself the basis for the more gel-like texture of the nucleus. Moreover, it is this increased water binding capacity provided by the proteoglycans in the nucleus that is critical to the compressive strength of the spine [30]. At the same time, the actual amount of loading placed on the spine likely plays a role in proteoglycan production and activity within the disc, suggesting, as with muscle tissue growth or atrophication, a transactive relationship between the body and its physical environment that is mediated, in large part, by the process of mechanotransduction. In short, the growth and maintenance of IVDs, as well as their healthy function and pathological degeneration, involve a complex interplay between mechanical, electrochemical and biochemical forces, with mechanotransduction providing the means by which external mechanical forces are translated into biochemical activity and processes within cells.

## The effects of mechanical stress on IVDs

Various studies have already shown that mechanical stress affects gene expression and metabolism in IVD cells both *in vivo* and *in vitro* [31–34], and as the preceding discussion should make clear, such mechanical stimulation can have both positive and negative effects on the health of IVDs.

**Fig. 4:**
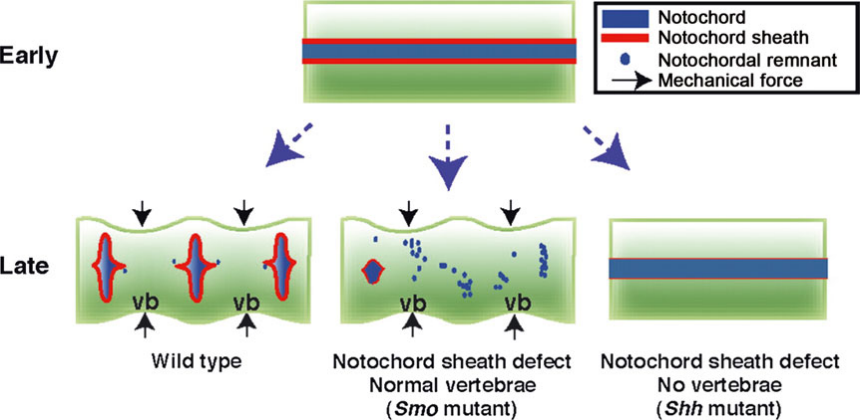
An illustration of the proposed role of the notochord sheath in the formation of the nuclei pulposi of intervertebral discs. The notochord sheath (indicated by the red line) starts to form, at E10.0 (early), around the notochord (blue line). By the E14.5 (late) time-point, the majority of the notochord cells are located within the intervertebral discs. One hypothesis is that swelling pressure (denoted by the arrows) from the vertebral bodies effectively pushes the notochord cells into the spaces between vertebrae. The notochord cells become randomly placed throughout the vertebral column, and small unshapely nuclei pulposi are formed following the loss of the functional sheath around the notochord (denoted by a thin red line). In the absence of any swelling pressure, functional sheath loss results in the rod-like notochord continuing to be present throughout the development of the embryo. The hypothesized model does not rule out the possibility that the movement of the notochords into the forming discs is the result of activity by some as yet unknown chemical or molecular pathway. (Figure adapted from Choi KS, Harfe BD. Hedgehog signaling is required for formation of the notochord sheath and patterning of nuclei pulposi within the intervertebral discs. *PNAS*. 2011; 108: 9484–9.)

Mechanical forces are critical for disc formation during embryogenesis. As demonstrated by recent research, for example, the notochord cells required for normal disc formation in mice and humans are likely pushed into the IVDs during embryogenesis by a mechanical force spurred by the formation of the vertebral bodies (Fig. 4) [35]. In contrast, in a study examining the effects of *in vivo* mechanical forces on human lumbar discs, Rajasekaran *et al*. used discs from patients with idiopathic adolescent scoliosis as a biological model to determine how tensile stress and compression affect disc health. Their results indicated that such stress can lead to decreased water content and cell density, matrix degeneration and calcification, and site-specific breaks in scoliotic discs [36].

**Fig. 5:**
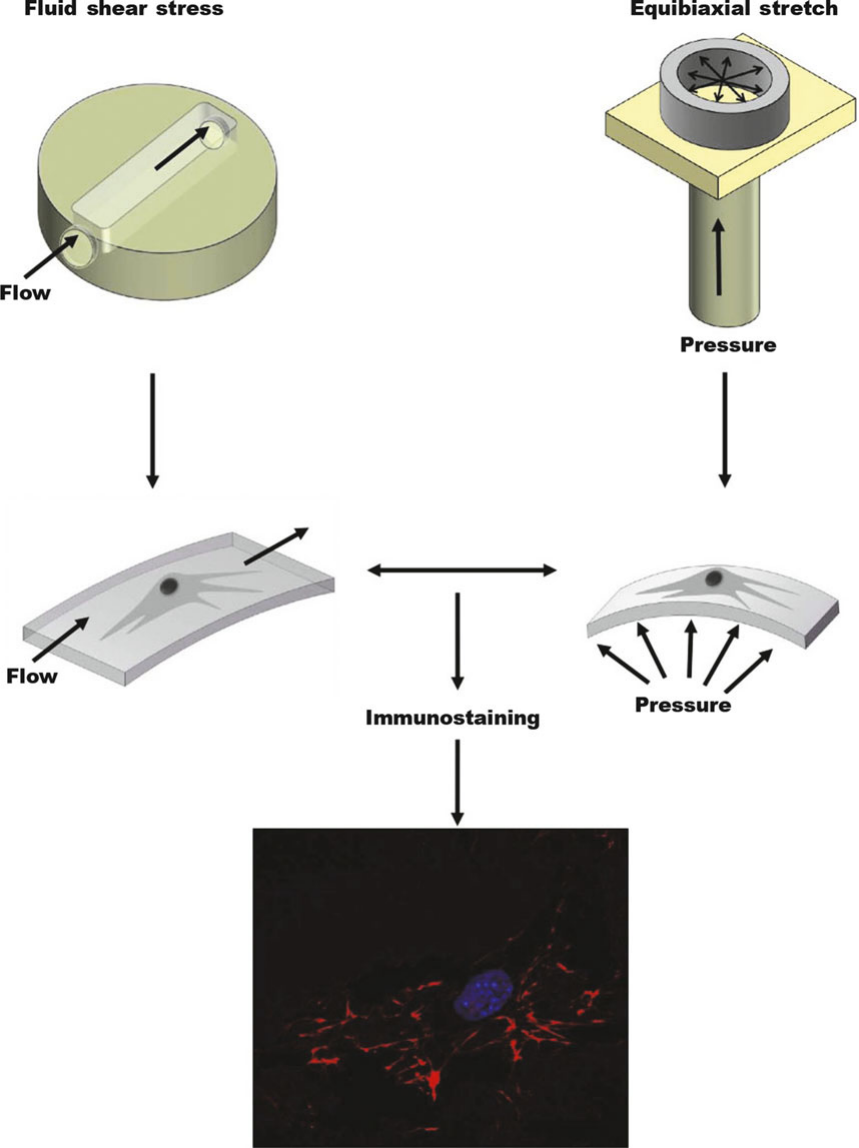
Schematic diagram of a system used to mechanically apply equibiaxial stretching and shear stress to living cells. The system consists of a pressure regulator used to exert a hydrostatic pressure-induced equibiaxial strain on the basal surface of cells and a peristaltic flow pump used to apply a shear stress across the apical surface of cells cultured on polydimethylsiloxane (PDMS). Immunostaining was then used to visualize FN following this mechanical stimulation. (Figure adapted from Steward RL, Cheng CM, Ye JD, *et al*. Mechanical stretch and shear flow induced reorganization and recruitment of fibronectin in fibroblasts. *Sci Rep*. 2011; 1: 147.)

As noted above, IVDs are affected by a wide variety of mechanical stimuli including shear stress, hydrostatic pressure, torsion, flexion and electrokinetic changes [37–39]. For example, Steward *et al*. recently introduced a device based on elastomeric substrates that allowed researchers to test the effects of shear fluid flow and uniaxial strip stretching on actin cytoskeleton (a key structural component of IVDs) and cell orientation. They found that the cytoskeleton and cells began to align themselves in the direction of the applied force after only 3 hrs of application [40]. A similar study also demonstrated how shear fluid flow and equibiaxial stretching influence fibroblast recruitment of fibronectin (another structural element of IVDs), with mechanically perturbed cells exhibiting increased fibronectin fibril formation and fibronectin localization at their peripheries (Fig. 5) [41].

Such studies are of great interest in terms of understanding mechanotransduction in general. However, for the purposes of the present review—considering mechanotransduction in IVDs—it will suffice to focus more closely on two of the most important forces affecting the spine: tensile stress and compression.

## Cyclic tensile stress *versus* compressive stress

Tensile stress is the form of stress caused by pulling forces (resulting in the lengthening of the stressed material in the direction of the applied force), and is one of the main types of stress that is regularly applied to IVDs and the spine in general. Cyclic tensile stress refers simply to tensile stress that changes over time in a repetitive fashion. Compression, on the other hand, consists of inwardly directed (or ‘pushing’) forces that decrease the length of an object in the same direction as the force applied; gravity is one major example of a compressive force applied to the spine. Like cyclic tensile stress, dynamic compression refers to any compressive force that changes over time in a repetitive fashion (as opposed to static compression).

In the spine, the annulus fibrosus provides the primary resistance to tensile stress and the nucleus pulposus provides most of the resistance to compressive forces [42]. In fact, when a compressive force is applied to the nucleus pulposus, the nucleus is deformed, resulting in the application of tensile force to the surrounding annulus fibrosus and endplates. So, even though the two types of forces effectively occur in conjunction, the nucleus pulposus absorbs the compressive force, while the annulus fibrosus dissipates the secondary tensile force.

The two types of cells also respond differently to the application of cyclic tensile strain. For example, when cyclic tensile strain was applied *in vitro* to bovine nucleus pulposus and annulus fibrosus cells, the majority of observable responses—including, for example, inhibition of MMP expression and increased type I collagen transcription—occurred only in the annulus pulposus cells, a fact that likely reflects the different forces encountered by the two different cell phenotypes *in vivo* [43].

The positive or negative effects of tensile strain on IVDs are highly dependent on the circumstances and the specific cell types to which they are applied. For example, whereas the Rajasekaran *et al*. study noted above found a variety of negative effects because of tensile stress applied to scoliotic discs, a study by Sowa and Agarwal demonstrated that tensile stress applied to fibrochondrocytes isolated from the annulus fibrosus of rats actually has a protective effect under conditions of inflammation [44]. Specifically, they showed that exposing cells to tensile stress at the same time as they were exposed to an inflammatory stimulus moderated the inflammatory response by decreasing cellular expression of the catabolic mediators of inflammation. A study by Gilbert *et al*., meanwhile, demonstrated further how different cell types and different stimuli can lead to highly divergent outcome. Specifically, they found that the responses of annulus fibrosus cells to cyclic tensile strain depend both on the frequency of the strain applied and whether or not the annulus fibrosus cells are derived from degenerated or non-degenerated tissue [45]. For example, cyclic tensile strain at a frequency of 1.0 Hz caused annulus fibrosus cells from non-degenerated tissue to decrease the expression of catabolic genes, whereas cyclic tensile strain at a frequency of 0.33 Hz caused the same type of annulus fibrosus cells to increase matrix catabolism.

As for compression, recent advances in the study of compression-induced mechanotransduction include the use of some of the elastomeric substrate techniques noted above. For example, Cheng *et al*. applied compression to cells on various PDMS substrates to determine its effects on cell–substrate interactions and morphological changes, and found that compression caused the overall cell structure, including the actin cytoskeleton, to reorient in the direction of the compression applied [46].

The above results go some way towards demonstrating the complexity and sensitivity of IVD cell responses to varying conditions. As another example, the effects of two factors alone, that is the age of the intervertebral cells and the frequency of the dynamic compression applied, have demonstrated dependence on variations in one another, with more mature cells responding quite differently (in terms of cellular phenotypes, rates of biosynthesis, and the production and maintenance of ECM components) to different compression frequencies than less mature cells [47]. At the same time, these findings and those of related studies suggest that mechanotransduction itself may have different pathways of activation depending on conditions as discrete as, say, the specific frequency of the applied tensile or compressive strain [48]. In any case, as complex as these interactions are, further studies will continue to illuminate their intricacies, providing important insights to improve our understanding of mechanotransduction, disc degeneration and potential therapies to repair or prevent such degeneration.

## Mechanotransduction in healthy and degenerated discs

As noted in the introduction, the means by which the above mechanical forces cause their most important effects on various cells is mechanotransduction. Without mechanotransduction, a compressive force would, for example, cause the nucleus pulposus of a disc to be physically deformed by a decrease in its height, but a whole host of critical intracellular changes in terms of gene expression, protein synthesis and even proliferation would not occur. In a sense, the capacity for mechanotransduction is one of the qualities that distinguish living tissues from most inanimate substances (although various types of synthetic mechanotransduction have been engineered in certain man-made products).

## Mechanoreceptors in IVDs

**Fig. 6:**
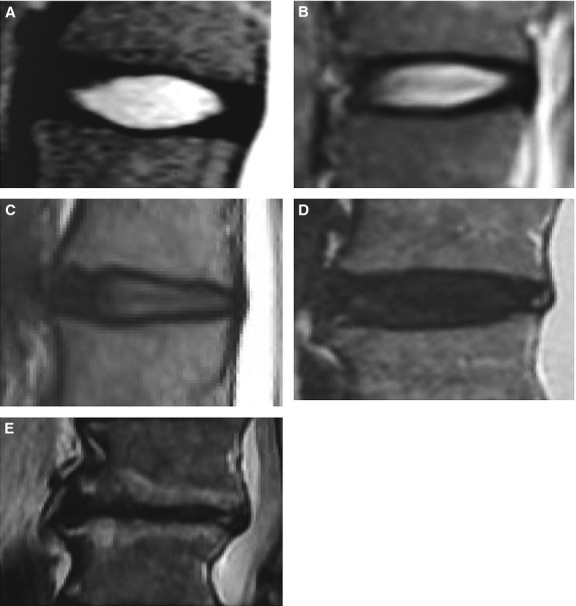
(**A**–**E**) Example images to illustrate a proposed grading system assessing lumbar disc degeneration. (**A**) Grade I: the disc structure is homogeneous, of normal height, and exhibits a hyperintense bright white signal. (**B**) Grade II: the disc structure is non-homogeneous with a hyperintense white signal. The disc height is normal, and the demarcation between annulus and nucleus remains clear, with or without grey horizontal bands. (**C**) Grade III: the disc structure is non-homogeneous, with a signal intensity of intermittent grey colour. The disc height is either normal or slightly decreased, with an unclear demarcation between nucleus and annulus. (**D**) Grade IV: the disc structure is non-homogeneous, with a hypointense signal intensity of dark grey colour. The disc height is normal or moderately decreased, and the demarcation between the nucleus and annulus is non-existent. (**E**) Grade V: the disc structure is non-homogeneous, with a hypointense black signal intensity. The disc space is collapsed, and the demarcation between nucleus and annulus is non-existent. Using this system, grading is conducted based on mid-sagittal fast spin-echo images that are T2-weighted.

Mechanoreceptors facilitate the transmission of mechanotransductive forces to cellular biochemical actions. Localized in nerve terminals, they allow tissues to respond directly to touch, vibration and pressure, among other physical stimuli [49]. Mechanoreceptors begin the biological response to mechanical stress through the firing of action potentials. Various types and concentrations of mechanoreceptors are found in different parts of the body. A study of sequential sections of human and bovine spines found that the mechanoreceptors in the annulus fibrosus and longitudinal ligaments consisted primarily of Pacinian corpuscles, Ruffini endings and, most frequently, Golgi tendon organs. It is suggested that the Pacini and Ruffini endings are primarily related to posture, while the Golgi tendon organs are related to pain. In this connection, they noted that Golgi tendon organs were found in differing percentages of discs from patients with scoliosis (15%) and lower back pain (50%) [50]. In a later study using magnetic resonance imaging to conduct a more direct comparison of mechanoreceptors in healthy and degenerated discs, Oliveira *et al*. found that mechanoreceptors play critical roles in different phases of disc degeneration, with a greater number of mechanoreceptors being found in degenerated discs than in normal discs [51]. With this in mind, the authors suggest that magnetic resonance provides a highly efficient means of detecting even the early-phases of disc degeneration, even as other authors have pointed out that the precise implications of these initial changes remain obscure [52]. Figure 6 illustrates one proposed schema for using magnetic resonance imaging to assess the degree of lumbar disc degeneration [53].

## Gene expression in IVDs

One of the primary means by which mechanoreceptors propagate the chain of mechanotransductive effects in cells is by altering intracellular levels of gene expression. This has direct effects on all the fundamental activities within and between cells, including proliferation, apoptosis and protein and enzyme synthesis, among others.

Accordingly, close examination of gene expressions under varying circumstances can elucidate the means by which mechanotransduction occurs under different circumstances. For example, Le Maitre *et al*. used dynamic compression combined with an RGD inhibitory peptide to demonstrate that nucleus pulposus cells from degenerated discs have a different mechanotransduction pathway than cells from healthy discs [54]. More specifically, they showed that RGD integrins were responsible for mechanosensing in the non-degenerated disc cells, while cells from degenerated discs exhibited a different signalling pathway (possibly, the authors suggest, one involving calcium and/or nitric oxide) that excluded those integrins.

In this connection, we noted above that recent research had identified proteins other than integrins that can also initiate mechanotransduction, a phrasing which actually indirectly suggests just how central integrins are to mechano-response; that is. prior to this more recent research, earlier studies had led researchers to view integrins as the primary transmembrane means of starting mechanotransduction within cells. For example, a finding regarding the role of integrins in annulus fibrosus cells similar to that which Le Maitre *et al*. reported in nucleus pulposus cells was recently detailed by Gilbert *et al*.: specifically, they found that, while RGD integrins do mediate the mechano-response of non-degenerated annulus fibrosus cells to cyclic tensile strain, these integrins are excluded from the same mechanotransduction pathway in annulus fibrosus cells from degenerated discs [55]. To sum the two studies up, RGD integrins mediate signalling pathways in both nucleus pulposus and annulus fibrosus cells from healthy discs, but they somehow become excluded from the mechanotransduction pathways in the same types of cells derived from unhealthy discs. Such studies demonstrate with increasing precision how mechanotransduction pathways become altered in degeneration and, in doing so, may lead to new options for treating IVD disease.

One of the key means by which integrins transmit their effects on transduction is *via* their connections to the cytoskeleton. Wang and Ingber used magnetic twisting to demonstrate the close continuity between cytoskeleton and integrins, showing that the latter are firmly attached to the former [56]. Furthermore, Chen *et al*. showed how the application of such twisting to integrin receptors results in increased endothelin-1 gene expression [57]. In fact, a variety of proteins, including tensin, talin and filamin, among others, have been shown to include binding domains for both cytoskeleton and integrins, providing various means for their direct physical linking [58].

Cytoskeletal elements themselves (*e.g*. microtubules and the Golgi apparatus) are directly affected by mechanical forces. For example, Hall *et al*. demonstrated that when hydrostatic pressure on cells becomes too high, these elements can become disorganized, hindering both protein synthesis and transport across the cell membranes [59]. At the same time, the cytoskeleton can undergo reorganization in response to hypo-osmotic conditions [60], a finding that is directly relevant to degeneration in IVDs, precisely because osmotic pressure changes as a result of decreased water content are known to be among the most profound changes that occur in IVDs as individuals go through the process of aging. Along these lines, a recent study by Tan *et al*. used synthetic cellular nanosystems to demonstrate how macromolecular crowding resulting from osmotic pressure can effectively be used, along with the adjustment of key variables such as the size of the crowding molecules, to control gene expressions in both natural and synthetic cellular systems [61]. Such cutting-edge research offers additional possibilities for exploring the effects of osmotic pressure on mechanotransduction involving various cell structures, including cytoskeletal elements in IVDs.

The structural integrity of the cytoskeleton is fundamental to the signal transduction induced by mechanical stimuli. For example, Pavalko *et al*. demonstrated that the application of cyclic forces to the cytoskeleton results in stronger protein anchorage that leads to increased activation of MAPKs [62]. Activated MAPKs themselves are the key to regulating the expression of various genes through phosphorylation of downstream transcription factors [63]. In the context of nucleus pulposus cells, MAPK pathways have recently been shown to regulate the expression of c-fos, which is itself an important factor in the pathogenesis of osteoarthritic joint disease [15]. In fact, Figure 2, our earlier example of the complexity of a mechanotransduction pathway, shows a model (one which we now hope readers will find easier to understand) of how this signalling pathway regulates c-fos in nucleus pulposus cells in the context of both disc degeneration and regeneration.

## Conclusion

As a result of the highly complex manner in which it occurs in living systems, mechanotransduction is not yet fully understood. Nonetheless, by building on the work of earlier experiments and by continuously fashioning new and/or more refined methods of investigating the process, researchers are learning more about the causes, effects and differences in mechanotransduction as it occurs under different circumstances and in different tissue types. In doing so, they are also able to test and learn more about potential therapies for various pathologies. With regard to IVDs, for example, there are a variety of potential therapies that might be used to prevent or reverse disc degeneration, including protein injection, gene transfer and cell implantation [64]. As our understanding of mechanotransduction in IVDs increases, the viability of these and other potential therapies will become more clear and may even, because of further refinement, be likewise increased.
